# Editors’ Note

**DOI:** 10.5195/ijt.2018.6270

**Published:** 2018-12-11

**Authors:** ELLEN R. COHN, JANA CASON

## ISSUE OVERVIEW

### POLICY

This issue begins with a 50-state survey of current policy affecting occupational therapy (OT) and physical therapy (PT). Authors Bierman, Kwong, and Calouro of The Center for Connected Health Policy presents methodology and analyses worthy of emulation by other healthcare professions. Regulatory-based professional uniformity within and across states are worthy attributional goals, both to facilitate the use of single-profession based telerehabilitation and to support team-based inter-professional practice.

### CLINICAL APPLICATIONS

The five articles that follow address the clinical applications of telerehabilitation. Each examines the contributions of telerehabilitation in a unique clinical circumstance. Two of five articles explore providers’ experience and perspectives. The evaluation of the benefits of telerehabilitation to avoid missed appointments will resonate across most healthcare professions. Finally, the use of telerehabilitation to address chronic spinal pain has great promise to ameliorate suffering and avoid more aggressive physical management.

## ACKNOWLEDGEMENTS

IJT is grateful to new and returning reviewers; William E. Janes, OTD, MSCI, OTR/L, Section Editor; colleagues at the Rehabilitation Engineering Research Center on Information and Communication Technology Access at the University of Pittsburgh; and, the IJT publishers at the University Library System at the University of Pittsburgh, especially Vanessa Gabler, Electronic Publications Manager.

## CALL FOR SUBMISIONS

We cordially invite submissions to the Spring 2019 issue by mid-February 2019. IJT accepts original research, case studies, viewpoints, technology reviews, book reviews, and country reports that detail the status of telerehabilitation.

Sincerely,

Ellen R. Cohn, PhD, CCC-SLP, ASHA-F, IJT Editor

Jana Cason, DHSc, OTR/L, FAOTA, Senior Associate Editor

